# Development and validation of a deep vein thrombosis risk assessment tool for surgical patients aged 75 years and older

**DOI:** 10.1186/s12877-026-07277-1

**Published:** 2026-03-05

**Authors:** Heqing Ye, Jingru Li, Libing Du, Chengtai Li, Rongzhu Chen

**Affiliations:** Department of Operating Room, The First Affiliated Hospital of USTC, Division of Life Science and Medicine, University of Science and Technology of China, Hefei, Anhui Province 230001 China

**Keywords:** Deep vein thrombosis, Predictive model, Older surgical patients, Nomogram

## Abstract

**Background:**

Deep vein thrombosis (DVT) is a common and severe medical condition characterized by the formation of thrombi in deep veins, primarily affecting older surgical patients. The present study aimed to identify risk factors for DVT in surgical patients aged 75 years and older and subsequently develop and validate a risk assessment tool for this patient population.

**Methods:**

A retrospective study was conducted on surgical patients (*n* = 686) aged 75 years and older at a tertiary general hospital in Hefei, China, from January to December 2024. Predictors for the model were selected using Least Absolute Shrinkage and Selection Operator (LASSO) regression, followed by multivariable logistic regression. Area under the curve, calibration curve, and decision curve analysis (DCA) were used to examine the discriminative power, calibration, and clinical efficacy of the predictive models. Internal validation was performed using both bootstrap resampling and 10-fold cross-validation.

**Results:**

The incidence of DVT among surgical patients aged 75 years and older was 14.7% (*n* = 101/686). Six predictors were identified and used to establish a nomogram: malignancy (OR: 7.590, 95% CI: 2.670–21.500), sex (OR: 0.387, 95% CI: 0.195–0.724), anesthesia duration (OR: 1.010, 95% CI: 1.006–1.014), D-dimer (OR: 1.210, 95% CI: 1.130–1.310), platelet count (OR: 1.010, 95% CI: 1.005–1.015), and pneumatic tourniquet application (OR: 2.700, 95% CI: 1.470–5.170). The nomogram demonstrated excellent discrimination (AUC = 0.786, 0.786 (95% CI, 0.738–0.834) and good calibration (Hosmer-Lemeshow test, *P* = 0.588). Upon interval validation, the model achieved a concordance index (C-index) of 0.791 (95% CI, 0.780–0.800). Finally, DCA demonstrated the net clinical benefit of this nomogram.

**Conclusions:**

This study constructed a practical model to predict DVT in surgical patients aged 75 years and older. This model incorporates demographic characteristics and clinical risk factors, enabling individualized prediction.

## Introduction

Deep vein thrombosis (DVT) is a prevalent perioperative complication [1] characterized by the formation of thrombi within the deep veins, usually of the lower limbs, causing partial or complete blockage of the venous lumen.

The global older population is projected to double to 1.5 billion by 2050 [2], with China facing significant aging challenges. Advanced age is an independent risk factor for DVT [3]. As age increases, the vascular elasticity of older adults significantly decreases, coupled with limited mobility, leading to slow blood flow in the lower limbs. Intraoperative and postoperative bleeding further places the blood in a state of hypercoagulability, ultimately resulting in DVT. Studies have shown that the incidence of DVT among older surgical patients is significantly higher than in the general population (approximately 12.3–57%) [4–6]. In a cohort study of over 90,000 residents of a Norwegian district, incidence rates for venous thromboembolism (VTE) and isolated DVT in individuals aged 70 years or older were more than three times as high as in those aged 45–69 years [7]. Current evidence suggests that patients who develop DVT have prolonged hospital stays, increased medical costs, and may also experience disability or death [8]. However, DVT is preventable. It is widely thought that appropriate preventive and therapeutic measures can reduce the incidence of postoperative lower extremity DVT by over 50% [9]. Contemporary perioperative care increasingly emphasizes multimodal prophylaxis, integrating chemoprophylaxis with mechanical devices and early mobilization. However, the efficacy and safety of these strategies are highly dependent on accurate risk assessment [10]. Currently, the Caprini risk assessment is the most widely used tool in surgery. Despite its comprehensive nature, this model requires extensive and expensive laboratory tests that may not be universally accessible, thereby limiting its applicability [11]. Recent research indicates that specialized risk assessment models tailored to specific surgical populations may exhibit superior predictive efficacy compared to the general Caprini model. A prospective multicenter cohort study conducted involving postoperative patients with colorectal cancer revealed that the CRC-VTE score, designed specifically for this population, demonstrated exceptional predictive performance for postoperative VTE, with an AUC of 0.72. This score significantly outperformed the Caprini model, which had an AUC of 0.59 [12]. The finding suggest that the general Caprini model may lack sufficient sensitivity to accurately assess VTE risk characteristics in specific populations.

Moreover, DVT risk prediction models for older surgical patients predominantly concentrate on orthopedic procedures, as well as genitourinary and colorectal cancer surgeries [13–16]. While these models demonstrate high predictive accuracy for patients undergoing these specific types of surgery, their accuracy may diminish when applied to other surgical populations. Given the rising life expectancy, it is imperative to develop a comprehensive risk assessment tool for DVT specifically tailored for older surgical patients, particularly those aged 75 and older.

Therefore, the present study sought to construct a DVT prediction model by determining the risk factors for DVT in surgical patients aged 75 years and older. The model was designed to aid clinicians in the timely identification of high-risk patients and offer tailored guidance for the prevention and treatment of DVT.

## Methods

### Participants and sample size

This retrospective study collected data on patients from hospital information systems. In this study, older surgical patients were recruited from a tertiary general hospital in Hefei, China, between January and December 2024, using convenience sampling. Patients were eligible for inclusion if they were aged 75 years or older and were scheduled to undergo a surgical procedure. Individuals were excluded if they had a preoperative diagnosis of DVT, a known coagulation disorder, or a history of severe cardiovascular or cerebrovascular disease. Riley et al. [17] proposed a four-step method for estimating sample size. In this study, the pmsampsize package in R was used to implement the sample size estimation based on this method. The sample size calculation was based on the following parameters: an R² of 0.2 (csrsquare = 0.2), 18 predictor variables (parameters = 18), and an event rate of 0.35 [6] (prevalence = 0.35). The sample size was determined using the pmsampsize package based on four criteria: Step 1 requires 540 participants to control overfitting; Step 2 requires 617 participants for stable predicted values; Step 3 requires 180 participants for precise intercept estimation; Step 4 requires 49 participants for precise R² estimation. The largest requirement, 617 participants, is the final sample size needed to ensure model calibration and prediction stability. A total of 686 patients were finally included, meeting this requirement.

### Diagnostic criteria for DVT

All patients received routine ultrasound screening on the first day after surgery. If DVT was suspected due to new limb swelling, pain, or redness, a confirmatory lower limb compression ultrasound was conducted immediately. Follow-up checks were done until postoperative day 7. DVT diagnosis followed established criteria [18]. The diagnostic criteria included the following ultrasonographic findings: a solid mass of uneven echogenicity in the lower extremities, with diminished or absent color flow and spectral signals, and non-compressibility of the venous lumen under probe pressure.

### Data collection

Demographic and clinical data were collected from all participants in the study. To improve the feasibility of the model, variables that were readily available in the clinic were selected, including: (1) general information (age, sex) and lifestyle habits (history of smoking, consumption of alcohol); (2) comorbid medical conditions (hypertension, diabetes mellitus (DM), malignancy); (3) laboratory tests of biochemical markers (hemoglobin (HB), prothrombin time (PT), D-dimer, C-reactive protein (CRP), platelet count (PLT), absolute neutrophil count (ANC), fibrinogen level (FIB), (4) anthropometric measurements (body mass index (BMI)); (5) surgical metrics (anesthesia duration (AD), intraoperative transfusion (IT), pneumatic tourniquet application(PTA)) .

### Statistical analysis

In the primary analysis, multiple imputations were employed to address missing data, with approximately 14.2%, 8.4%, and 1.1% of values missing for CRP, BMI, and D-dimer, respectively. All statistical analyses were performed using IBM SPSS version 23.0 and R 4.5.0. Continuous variables with non-normal distributions were expressed as medians (interquartile ranges, IQR), and the Mann-Whitney U test was used for comparison between groups. Count or categorical data were displayed as numbers (percentage, %) and analyzed with the Chi-square test or Fisher’s exact test for comparison.

The least absolute shrinkage and selection operator (LASSO) method was applied to screen for predictive factors. Statistically significant variables (*P* < 0.05) were included in the stepwise multivariate logistic regression analysis. Independent predictors identified by the multivariate logistic analysis were selected to establish a predictive nomogram.

The model’s performance was evaluated using calibration plots, decision curve analysis (DCA), and the area under the receiver operating characteristic curve (AUC). Internal validation was performed using the bootstrap method with 1000 iterations.

## Results

### General characteristics of the patients

After applying inclusion and exclusion criteria, a total of 686 older patients were enrolled in the study, exhibiting female predominance (*n* = 452, 65.8%) and a median age of 78 years (IQR: 76–81 years). The incidence of DVT in these patients was 14.7% (*n* = 101/686). Patient characteristics are detailed in Table 1.

**Table 1:** Baseline characteristics of included patients

Variables	Total (*n* = 686)	None-DVT (*n* = 585)	DVT (*n* = 101)	t/Z/χ²	*P*-value
Age, (median [IQR], years)	78.00 (76.00, 81.00)	78.00 (76.00, 81.00)	78.00 (76.00, 82.00)	-0.63	0.527
BMI, (median [IQR], kg/m^2^)	24.60 (21.83, 27.10)	24.60 (22.00, 27.20)	24.40 (21.20, 27.10)	-1.00	0.318
AD, (median [IQR], min)	105.00 (85.00, 125.00)	99.00 (85.00, 120.00)	110.00 (94.00, 155.00)	-4.04	< 0.001
PT, (median [IQR], s)	11.80 (11.30, 12.50)	11.80 (11.30, 12.50)	11.90 (11.30, 12.60)	-0.51	0.611
D-dimer, (median [IQR], µg/mL)	0.80 (0.44, 1.96)	0.73 (0.43, 1.65)	1.64 (0.69, 3.95)	-5.45	< 0.001
CRP, (median [IQR], mg/L)	3.11 (3.08, 13.50)	3.11 (3.08, 11.60)	4.80 (3.11, 32.20)	-3.05	0.002
HB, (median [IQR], g/L)	124.00 (113.25, 133.00)	124.00 (115.00, 134.00)	120.00 (106.00, 130.00)	-3.31	< 0.001
PLT, (median [IQR], 10^9/L)	201.00 (165.00, 245.75)	198.00 (164.00, 240.00)	221.00 (185.00, 279.00)	-3.73	< 0.001
ANC, (median [IQR], 10^9/L)	3.56 (2.70, 4.76)	3.53 (2.69, 4.68)	3.82 (2.76, 5.66)	-1.80	0.071
FIB, (median [IQR], g/L)	3.46 (2.89, 4.20)	3.43 (2.89, 4.11)	3.74 (3.05, 4.48)	-2.31	0.021
Malignancy, n(%)				30.39	< 0.001
No	654 (95.34)	569 (97.26)	85 (84.16)		
Yes	32 (4.66)	16 (2.74)	16 (15.84)		
Sex, n(%)				5.64	0.018
Female	452 (65.89)	375 (64.10)	77 (76.24)		
Male	234 (34.11)	210 (35.90)	24 (23.76)		
IT, n(%)				2.63	0.105
No	661 (96.36)	567 (96.92)	94 (93.07)		
Yes	25 (3.64)	18 (3.08)	7 (6.93)		
PTA, n(%)				0.50	0.481
No	331 (48.25)	279 (47.69)	52 (51.49)		
Yes	355 (51.75)	306 (52.31)	49 (48.51)		
Smoking, n(%)				0.90	0.343
No	657 (95.77)	558 (95.38)	99 (98.02)		
Yes	29 (4.23)	27 (4.62)	2 (1.98)		
Alcohol, n(%)				1.49	0.222
No	648 (94.46)	550 (94.02)	98 (97.03)		
Yes	38 (5.54)	35 (5.98)	3 (2.97)		
Hypertension, n(%)				2.24	0.135
No	313 (45.63)	260 (44.44)	53 (52.48)		
Yes	373 (54.37)	325 (55.56)	48 (47.52)		
DM, n(%)				2.31	0.128
No	554 (80.76)	478 (81.71)	76 (75.25)		
Yes	132 (19.24)	107 (18.29)	25 (24.75)		

t: t-test, Z: Mann-Whitney test, χ²: Chi-square test, *SD* standard deviation, *IQR* interquartile range

### Screening for predictive factors and construction of the nomogram for DVT

**Fig. 1:**
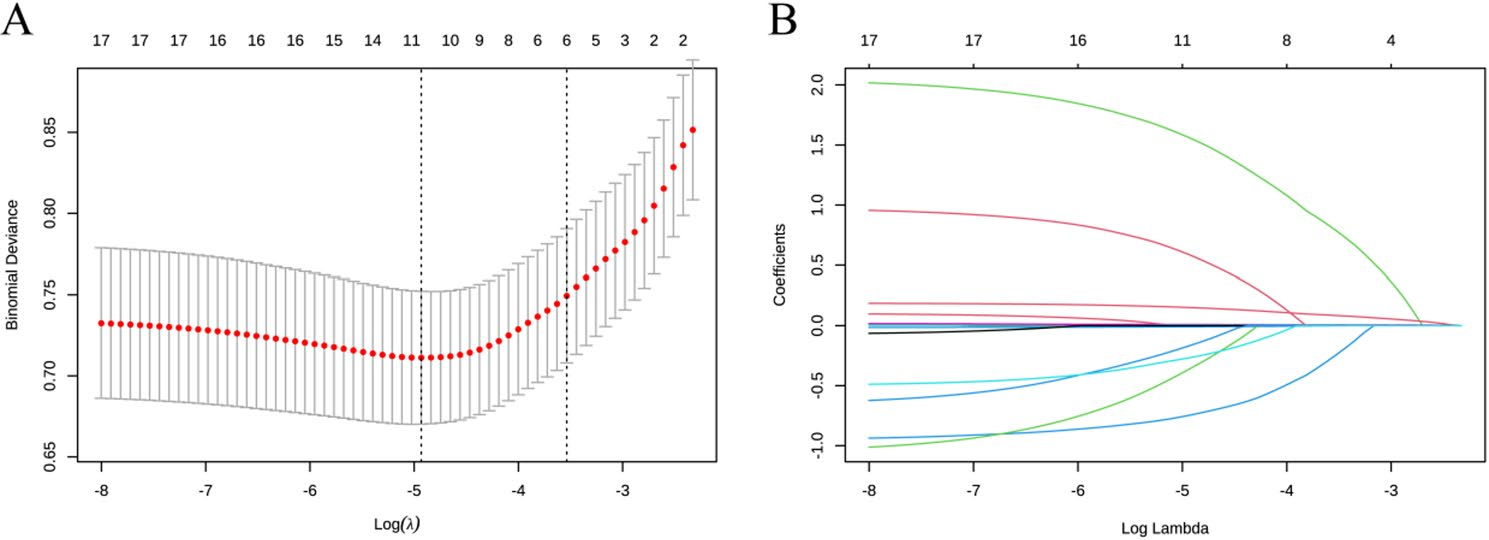
Data statistics and clinical feature selection using the LASSO binary logistic regression model. **A **Optimal parameter (lambda) selection in the LASSO model was conducted using 10-fold cross-validation based on the minimum criteria. The partial likelihood deviance (binomial deviance) curve was plotted versus log(lambda). **B **LASSO coefficient profiles for the 11 candidate features, plotted against the log(lambda) sequence

**Fig. 2:**
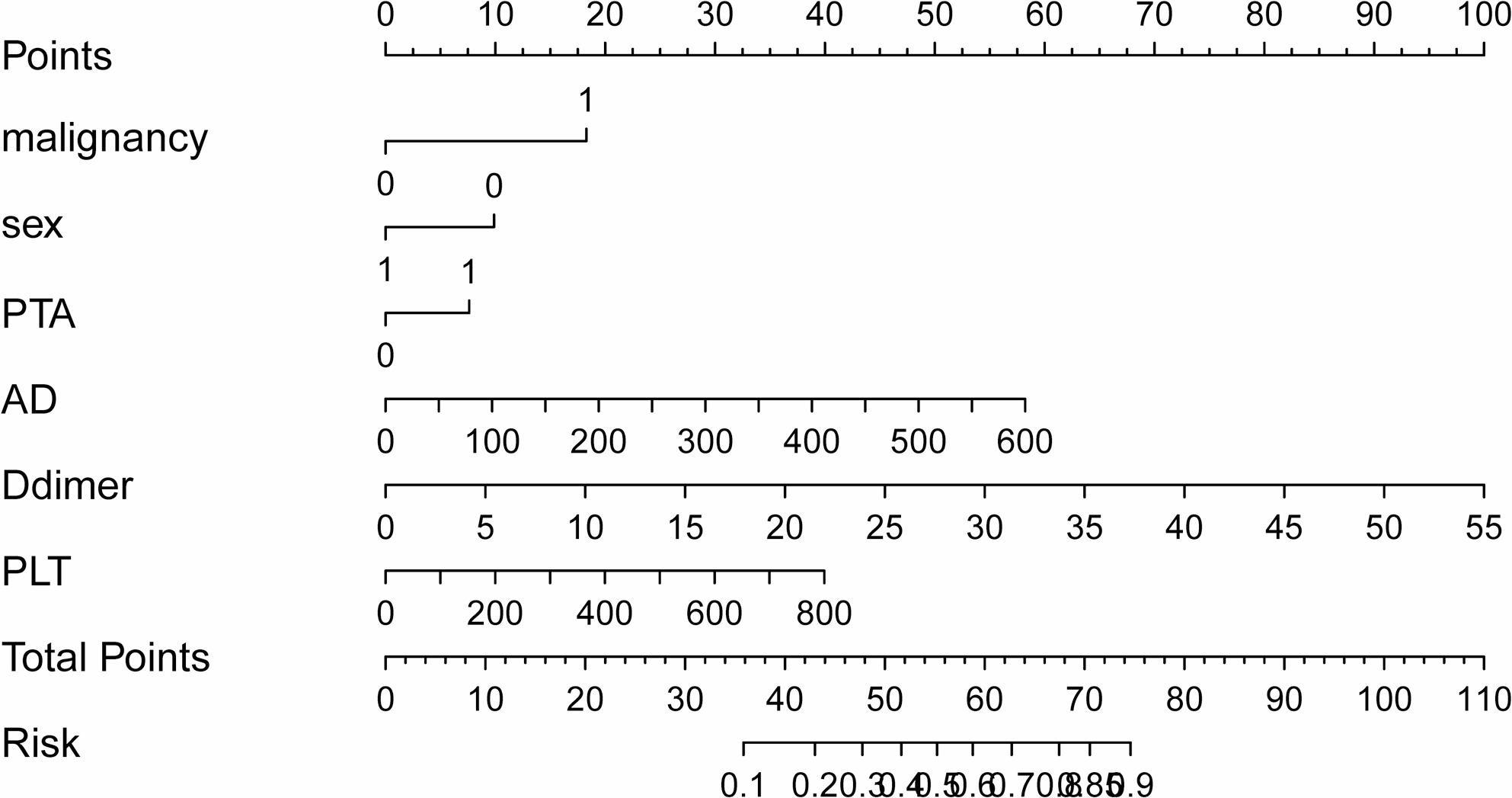
Nomogram for predicting the risk of DVT in older surgical patients

A tenfold cross-validation was employed to optimize the regularization parameter (λ), with the optimal λ selected at lambda.min = 0.0087 to ensure the model identifies more latent factors. At log(λ) = − 4.745, eleven non-zero coefficient features were identified: sex, CRP, D-dimer, PLT, HB, AD, hypertension, malignancy, history of smoking, consumption of alcohol and PTA (Fig. 1). The stepwise multivariate regression analysis identified malignancy (OR: 7.590, 95% CI: 2.670–21.500), sex (OR: 0.387, 95% CI: 0.195–0.724), AD (OR: 1.010, 95% CI: 1.006–1.014), D-dimer (OR: 1.210, 95% CI: 1.130–1.310), PLT (OR: 1.010, 95% CI: 1.005–1.015), and PTA (OR: 2.700, 95% CI: 1.470–5.170) as independent predictors of DVT (Table 2). These independent predictors were incorporated into a predictive model and visualized as a nomogram (Fig. 2). A sensitivity analysis has been conducted by repeating the primary analysis on the complete-case dataset (CCD). The findings from this sensitivity analysis aligned with those derived from the multiply imputed dataset, both in direction and magnitude of effect, thereby underscoring the robustness of our conclusions (Table 3).

**Table 2:** Multivariate logistic regression analysis of predictive factors for DVT

Variables	β	Se	Wald	Odds ratio	95% CI	*P* -value
Malignancy, n(%)	3.84	0.528	14.75	7.590	(2.670, 21.500)	< 0.001
Sex, n(%)	-2.85	0.333	8.12	0.387	(0.195, 0.724)	0.004
AD (min)	4.43	0.003	19.62	1.010	(1.006, 1.014)	< 0.001
D-dimer (ug/ml)	4.83	0.039	23.33	1.210	(1.130, 1.310)	< 0.001
PLT (10^9/L)	3.52	0.002	12.39	1.010	(1.005, 1.015)	< 0.001
PTA, n(%)	3.12	0.319	9.73	2.700	(1.470, 5.170)	0.002

The logistic regression model included an intercept term

**Table 3:** Comparison of sensitivity analysis results

Variables	Analysis type	Odds ratio	95%CI	*P*-value
Malignancy	MI	7.59	2.67–21.50	< 0.001
	CCD	9.41	1.86–45.49	0.009
D-dimer	MI	1.21	1.13–1.31	< 0.001
	CCD	1.33	1.19–1.50	< 0.001
PTA	MI	2.70	1.47–5.17	0.002
	CCD	3.10	1.56–6.60	< 0.001
Sex	MI	0.387	0.195–0.724	0.004
	CCD	0.340	0.150–0.690	0.002
PLT	MI	1.01	1.005–1.015	< 0.001
	CCD	1.01	1.00-1.01	0.003
AD	MI	1.01	1.006–1.014	< 0.001
	CCD	1.01	1.00-1.02	0.008

### Predictive performance and clinical utility of the nomogram

**Fig. 3:**
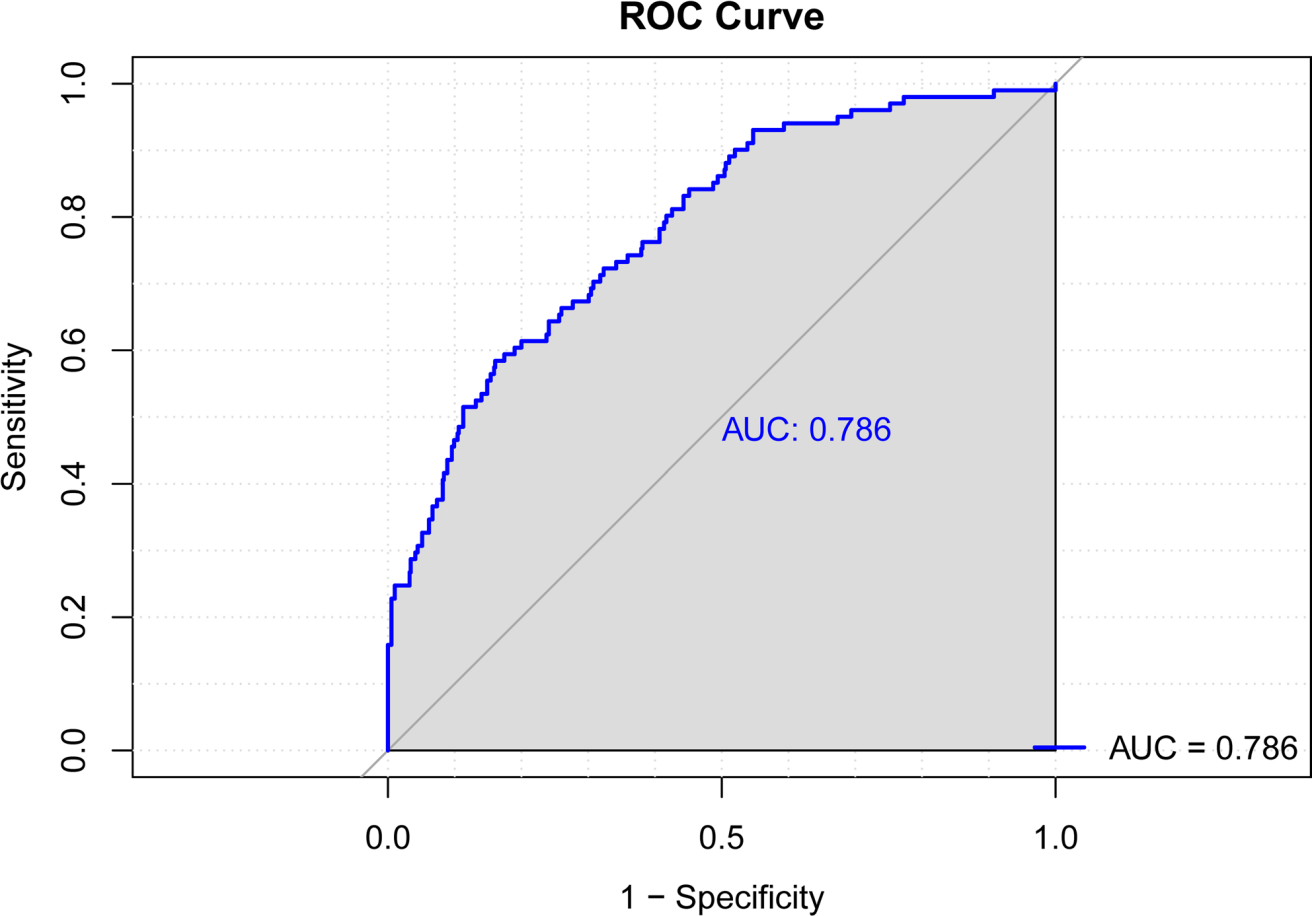
ROC curve analysis for the predictive values of DVT

**Fig. 4:**
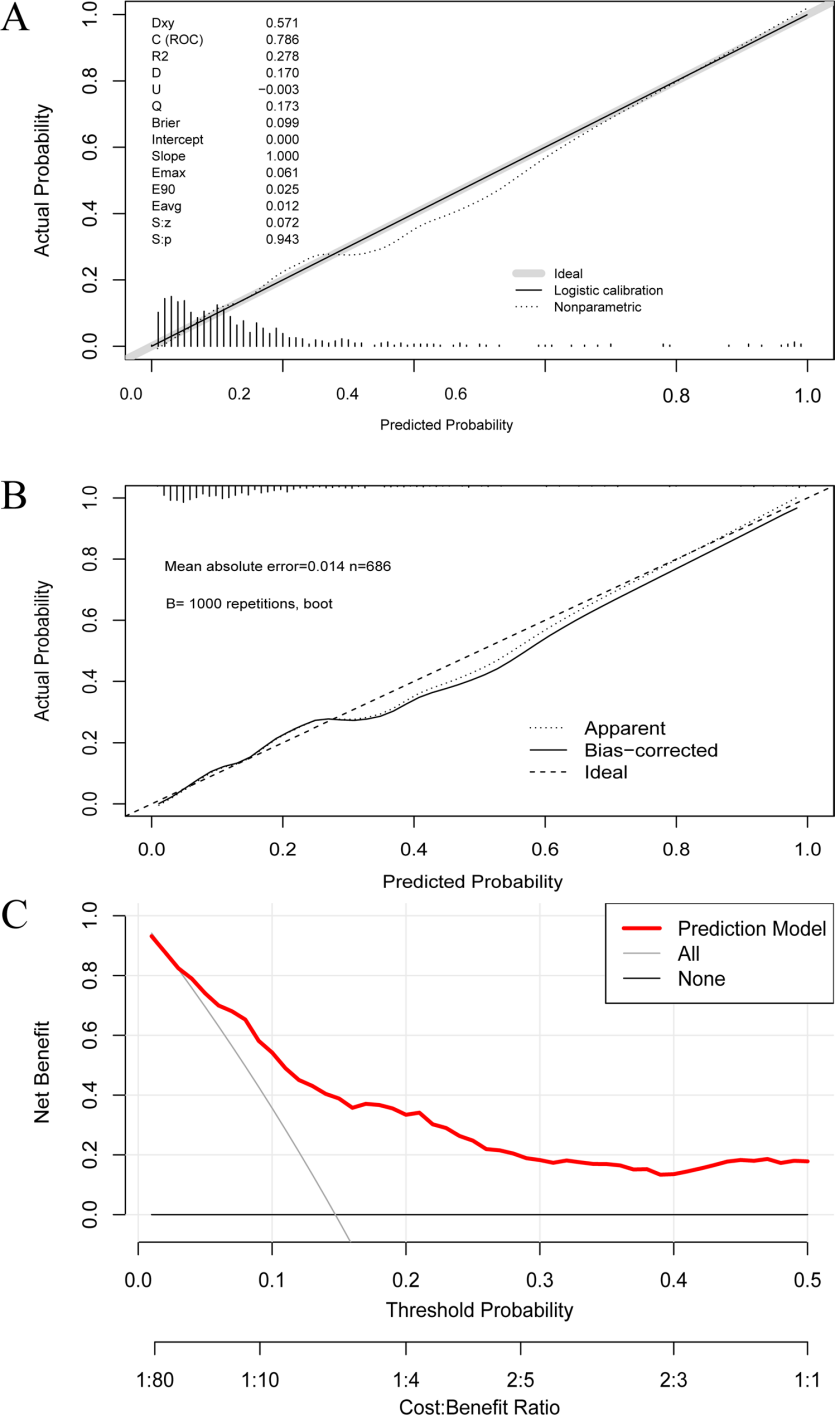
Calibration curves and decision curve analysis of the nomogram. **A** Calibration curves for the model development cohort. **B** Calibration curve from the internal validation. **C** DCA curve for the final nomogram

The nomogram demonstrated good discriminative ability, with an AUC of 0.786 (95% CI, 0.738–0.834) (Fig. 3). Calibration curves used for estimating DVT exhibited good agreement, with a *P* value of 0.588. The nomogram was validated by bootstrapping with 1000 resamples. The calibration plot exhibited strong agreement between predicted and observed outcomes, closely aligning with the ideal line. The bootstrap internal validation C-statistic was 0.791 (95% CI, 0.780–0.800), indicating good performance. The clinical utility of the model was evaluated by DCA. The results indicate that when the threshold probability ranges from 10% to 30%, the model provides greater net benefits (Fig. 4).

## Discussion

In this retrospective study, 18 clinical features were identified for their association with DVT in older surgical patients. Six key predictors, malignancy, sex, AD, D-dimer, PLT, and PTA, were selected using LASSO with ten-fold cross-validation. A nomogram based on these variables exhibited good performance in predicting DVT in surgical patients aged 75 years and older, yielding an AUC of 0.786.

Our research found that older female surgical patients experience a higher risk of DVT, which can be attributed to postmenopausal gene differences and hormonal changes, along with their related complications. The findings of Roach et al. [19] suggest that genetic factors can account for the gender differences in DVT risk. Gene mutations exhibiting sex-specific effects may contribute to differences in the incidence of initial and recurrent venous thrombosis.

In addition, older women are more likely to develop conditions like nephrosis, which significantly increase the risk of DVT [20]. These findings suggest that gender is an essential factor in predicting DVT risk. Future research will focus on sex-specific risks to clarify the underlying pathophysiology and enhance tailored treatment and prevention strategies. In terms of hormonal influences, existing evidence indicates that Menopause hormone therapy (MHT) in postmenopausal women is associated with an increased risk of VTE. Findings from a systematic review and meta-analysis demonstrated that the use of MHT among postmenopausal women is correlated with a higher risk of stroke and venous thrombosis (RR = 1.86, 95% CI : 1.39–2.50) [21].

It is well-established that malignancy contributes to the occurrence of DVT in older surgical patients, which was corroborated by our study. Tumor-induced hypercoagulability and inflammatory response are considered essential mechanisms for the occurrence of DVT [22]. Interestingly, a study reported that in patients with malignancy, DVT occurrence is closely related to elevated inflammatory markers such as CRP and D-dimer levels [23]. In addition, Du et al. reported that older patients undergoing surgery for abdominal malignancy experience a significantly increased risk of DVT [24]. Another study indicated that among patients undergoing robotic surgery, the incidence of deep venous thrombosis and pulmonary embolism was 5.0%, suggesting that even in minimally invasive surgery, patients with malignancy remain at high risk of thrombosis [25]. These studies provide important guidance for clinical practice, emphasizing the importance of DVT risk assessment and prevention in older surgical patients with malignancy.

The use of pneumatic tourniquets has raised widespread concern about their effect on the incidence of DVT in surgical patients aged 75 years and older. DVT is a common complication after surgery and is particularly significant in older patients. In a randomized controlled trial, researchers found that the incidence of distal DVT in the tourniquet group was significantly higher than in the control group without tourniquets (52.9% vs. 23.1%; *P* = 0.002) [26]. This finding aligned with prior literature documenting that patients who did not use tourniquets during anterior cruciate ligament (ACL) reconstruction experienced a significantly lower incidence of DVT compared to those who did [27]. A meta-analysis concentrating on total knee arthroplasty revealed no statistically significant difference in postoperative DVT risk between groups utilizing a tourniquet and those not using one [28]. Conversely, a recent meta-analysis identified the duration of tourniquet application as a significant risk factor for DVT in ACL reconstruction [29]. These findings imply that regulating the duration of tourniquet application could be advantageous in mitigating the risk of postoperative DVT.

Our research demonstrated that longer anesthesia duration increases the incidence of DVT in older surgical patients, which may be due to the release of tissue factors promoted by anesthesia and surgical trauma. The tissue factor activates the extrinsic coagulation system, leading to a hypercoagulable state and thereby triggering DVT [30]. Phan et al. found that patients with longer anesthesia duration experienced a significantly increased risk of complications, including VTE [31]. Therefore, optimizing anesthesia management may be a crucial strategy to prevent DVT in older patients.

D-dimer is a product of fibrin degradation that typically appears after blood clots dissolve. It is widely used to screen for DVT [32, 33]. Our research indicated that preoperative D-dimer levels are an independent risk factor for DVT in older surgical patients, consistent with the conclusions of Hang et al. [18]. Another study also found that 18% of those with elevated preoperative D-dimer developed DVT. This risk was pronounced for bladder cancer patients and older individuals who are inherently more susceptible to DVT [34]. Our study also demonstrated that elevated preoperative PLT is a risk factor for DVT. It is widely acknowledged that platelets represent a key cellular component in venous thrombus tissue, alongside fibrin and red blood cells [35, 36]. Gonzalez et al. conducted a study on patients with hip fractures and found that compared to those with normal PLT counts, patients with elevated preoperative PLT counts had a significantly increased probability of developing DVT [37]. Dave et al. also pointed out that an elevated preoperative PLT count is associated with an increased risk of DVT in older patients [38]. Consequently, during preoperative assessments, emphasis should be placed on patients with elevated D-dimer and PLT, and appropriate preventive measures should be implemented to reduce the risk of DVT.

## Limitations

This study employed a single-center retrospective design, which limits the generalizability of the findings. Although the nomogram underwent internal validation, the absence of external data compromises the model’s reliability. The retrospective nature of data collection resulted in the omission of several critical variables, such as the preoperative frailty index and anticoagulation therapy. Furthermore, the follow-up period was restricted to the first seven days post-surgery at our center, potentially underestimating delayed thrombotic events in elderly patients. Future research will involve multicenter, prospective studies to enhance the model’s generalizability and clinical robustness through external validation. Additionally, geriatric-specific variables will be incorporated, and the follow-up duration will be extended to continuously improve the model’s predictive performance.

## Conclusion

This study established and verified a nomogram model that can predict the risk of DVT in surgical patients aged 75 and older. Our nomogram model, which combines malignancy, sex, AD, D-dimer, PLT, and PTA, was verified internally as a useful tool for risk assessment. The developed predictive model will be valuable in screening patients aged 75 and older with at high risk for DVT.

## Data Availability

The datasets used and /or analyzed during this study are not publicly available due to confidentiality of patient information and ethical restrictions but are available in summary form from the corresponding author upon reasonable request.

## Abbreviations

DVT: Deep vein thrombosis
VTE: Venous thromboembolism
CRC: Colorectal cancer
DM: Diabetes mellitus
HB: Hemoglobin
PT: Prothrombin time
CRP: C-reactive protein
PLT: Platelet count
ANC: Absolute neutrophil count
FIB: Fibrinogen level
BMI: Body mass index
AD: Anesthesia duration
IT: Intraoperative transfusion
PTA: Pneumatic tourniquet application
MI: Multiple imputations
IQR: Interquartile ranges
LASSO: Least absolute shrinkage and selection operator
DCA: Decision curve analysis
AUC: The area under the receiver operating characteristic curve
CCD: Complete-case dataset
MHT: Menopause hormone therapy
ACL: Anterior cruciate ligament
